# Hepatoprotective Effects of Chinese Medicine Herbs Decoction on Liver Cirrhosis in Rats

**DOI:** 10.1155/2017/6125829

**Published:** 2017-02-09

**Authors:** Nor Aziyah Mat-Rahim, Tong-Hye Lim, Nur-Asyura Nor-Amdan, Sazaly AbuBakar

**Affiliations:** ^1^Tropical Infectious Diseases Research and Education Center (TIDREC), Department of Medical Microbiology, Faculty of Medicine, University of Malaya, 50603 Kuala Lumpur, Malaysia; ^2^Virology Unit, Institute for Medical Research, Jalan Pahang, 50588 Kuala Lumpur, Malaysia; ^3^Herbitec (M) Sdn Bhd, 11-2 Jalan Sri Hartamas 7, Taman Sri Hartamas, 50480 Kuala Lumpur, Malaysia

## Abstract

Hepatoprotective and curative activities of aqueous extract of decoction containing 10 Chinese medicinal herbs (HPE-XA-08) were evaluated in Sprague–Dawley albino rats with liver damage induced by thioacetamide (TAA). These activities were assessed by investigating the liver enzymes level and also histopathology investigation. Increases in alkaline phosphatase (ALP) and gamma-glutamyl transferase (GGT) levels were observed in rats with cirrhotic liver. No significant alterations of the liver enzymes were observed following treatment with HPE-XA-08. Histopathology examination of rats treated with HPE-XA-08 at 250 mg/kg body weight, however, exhibited moderate liver protective effects. Reduced extracellular matrix (ECM) proteins within the hepatocytes were noted in comparison to the cirrhotic liver. The curative effects of HPE-XA-08 were observed with marked decrease in the level of ALP (more than 3x) and level of GGT (more than 2x) in cirrhotic rat treated with 600 mg/kg body weight HPE-XA-08 in comparison to cirrhotic rat treated with just water diluent. Reversion of cirrhotic liver to normal liver condition in rats treated with HPE-XA-08 was observed. Results from the present study suggest that HPE-XA-08 treatment assisted in the protection from liver cirrhosis and improved the recovery of cirrhotic liver.

## 1. Introduction

Chronic liver disease is the ninth leading cause of mortality in Western and developing countries [1]. The disease resulted from chronic proinflammatory injuries, which could cause progressive fibrosis causing the liver to scar and eventually becomes cirrhotic. There are many factors that could lead to chronic liver injury and among them are viral infections such as hepatitis C infection, chronic alcoholism, and autoimmune diseases and also due to drug or substance abuses. Five percent of persons with liver cirrhosis could progress to develop liver cancer [2].

To date, there is no specific treatment for liver cirrhosis. Sufferers are treated to reduce the complications due to the damaged liver from exacerbating. The treatments are often expensive especially for those in developing countries where there is high rate of liver cirrhosis in the population. Due to these factors, treatment using ethnobotanical approach has gained popularity as an alternative cost-effective approach [1, 3]. Among the ethnobotanical approaches, the Chinese herbal medicine has been widely applied. It serves as alternative complementary medicine, probably due to the presence of complete pharmacopeia of the herbs established over more than 5000 years of traditional use. Currently, in many healthcare facilities in China, traditional Chinese medicine is being applied in complement to Western medicine [4]. Typical in most traditional Chinese medicines, the formulation consists of multiple herbs concoction. It has been suggested that the combination of herbs with the various combinations of natural ingredients may produce synergistic effects and neutralize potential side effects of an individual herb constituent, hence augmenting the effectiveness of a treatment [5–7].

The present study aims to evaluate the preventive and potential curative effects of a decoction containing 10 Chinese medicinal herbs, HPE-XA-08, in which a number of the herbs have been reported to be useful in supporting liver functions and help to ameliorate liver fibrosis/cancer [8–11].

## 2. Materials and Methods

### 2.1. Plant Extracts

Aqueous extract of HPE-XA-08 containing mixture of 10 Chinese medicinal herbs, comprising fructus* Gardenia jasminoides *Ellis,* Var. radicans* (Thunb.) Makino,* Artemisia scoparia* Waldst. Et Kit, radix* Rheum tanguticum* Maxim. Ex Balf, radix* Scutellaria baicalensis *Georgi, fructus* Ligustrum lucidum *Ait, cortex* Phellodendron amurense* Rupr.,* Poria cocoa *(Schw.) Wolf, radix* Bupleurum B. scorzoneraefolium *Willd, and flos* Chrysanthemum morifolium *Ramat and herbs* Tarxacum mongolicum *Hand, Mazz were prepared according to propriety extraction method of Herbitec (M) Sdn. Bhd. The aqueous extract was filtered to remove all the coarse residues, sterile filtered, and kept at −20°C until being needed for the study.

### 2.2. Animals

Male Sprague–Dawley (SD) rats aged between 7 and 10 weeks were kept in the University Malaya animal breeding house. Animals were fed with standard pellet diet and water ad libitum at 20–25°C. All animal handlings and protocols were in accordance with the institutional guidelines for laboratory animals (Ethic Reference Number PM/27/08/2011/MAA(R)).

### 2.3. Evaluation for Prevention and Treatment of TAA-Induced Liver Cirrhosis by HPE-XA-08

Preventive effects for liver cirrhosis conferred by HPE-XA-08 were evaluated using the SD rats. Rats were assigned to 4 groups, namely, Group 1P: control group 1 (normal saline + H_2_O), Group 2P: control group 2 (normal saline + HPE-XA-08), Group 3P: TAA control group (TAA+ H_2_O), and Group 4P: treated group (TAA + HPE-XA-08 treatment; pretreatment with HPE-XA-08, 300 mg/kg). Rats from Groups 1P and 2P were injected intraperitoneally (ip) with normal saline while Groups 3P and 4P were injected with thioacetamide (TAA) at a dose of 200 mg/kg twice weekly for 12 weeks. HPE-XA-08 was administered orally at 300 mg/kg via a stomach tube to rats in Groups 2P and 4P daily, up to 12 weeks, while Group 1P and Group 3P were fed with distilled water. For the evaluation of HPE-XA-08 treatment on TAA-induced liver cirrhosis, 3 rat groups were assigned, namely, Group 1T: normal control group, Group 2T: cirrhosis control group, and Group 3T: treatment group (posttreatment with HPE-XA-08, 600 mg/kg). Liver cirrhosis was induced in rats of Groups 2T and 3T by inoculating TAA at 200 mg/kg intraperitoneally, twice weekly, for 12 weeks. Rats of Group 1T that served as normal control were inoculated with normal saline. After 12 weeks, the TAA was stopped and the rats from Group 3T were treated with 600 mg/kg HPE-XA-08 as described above while rats of Groups 1T and 2T were treated with distilled water of the equivalent volume. Blood was collected from each rat before and after the treatment. The treatment was performed for 30 days.

### 2.4. Hepatic Biochemical Evaluation

Blood of each rat was collected via tail vein before the treatment and at the end of the treatment regime. The blood was collected in tube containing EDTA and analyzed for the liver function enzymes serum alanine transaminase (ALT), aspartate transaminase (AST), alkaline phosphatase (ALP), and gamma-glutamyl transferase (GGT) in the Clinical Diagnostic Laboratory (CDL) at University Malaya Medical Centre (UMMC). Immediately after the final blood collection, the rats were euthanized and the organs were harvested for histological investigation.

### 2.5. Histopathological Analysis

Liver samples harvested from the rats were washed with the normal saline and immediately fixed in 10% buffered neutral formalin for 48 hours. Samples were then embedded in paraffin wax. Sections of 5-micron thickness were prepared, processed in alcohol-xylene series, and stained with alum-haematoxylin and eosin, prior to histopathological examination.

### 2.6. Statistical Analysis

Results were presented as mean ± SEM of six animals in each group. The data were subjected to one-way ANOVA followed by Bonferroni's posttest. *p* < 0.05 was considered statistically significant. Analysis was performed using GraphPad Prism version 4.00 for Windows (GraphPad Software, USA).

## 3. Results

### 3.1. Prevention of TAA-Induced Liver Cirrhosis

The effects of HPE-XA-08 on serum ALT, AST, ALP, and GGT and bilirubin activities in rats from all treatment groups were shown in Table 1. It is observed that the serum ALT and AST activities in all rat groups, Groups 1P, 2P, 3P, and 4P, did not show any significant differences following the preventive treatment regime. It was noted that the serum ALT activity in rat group treated with HPE-XA-08 showed lower ALT activity with value of 76.0 ± 4.416 IU/L (Group 2P) and 69.40 ± 7.756 IU/L (Group 4P) in comparison to 80.0 ± 4.175 IU/L (Group 1P) and 88.5 ± 12.75 IU/L (Group 3P). For serum AST level, the TAA control group (Group 3P) showed the lowest level with value of 215.0 ± 13.64 IU/L, followed by Groups 4P, 2P, and 1P with values of 222.4 ± 5.715 IU/L, 223.3 ± 11.16 IU/L, and 237.3 ± 12.84 IU/L, respectively. Serum ALP level in rat groups injected with TAA (Groups 3P and 4P) were higher in comparison to those injected with normal saline only (Groups 1P and 2P). The highest level of ALP was observed in Group 3P with value of 375.0 ± 10.41 IU/L. The ALP level in rat group injected with TAA and treated with HPE-XA-08 (Group 4P) was observed to be lower than those treated with water (Group 3P) with value of 277 ± 4.722 IU/L. The serum GGT levels in rat groups injected with TAA (Groups 3P and 4P) were significantly higher in comparison to the control groups (Groups 1P and 2P). Serum GGT levels in TAA control group (Group 3P) were the highest with value of 37.50 ± 1.857 IU/L mmol/L. The rat group 4P (TAA + HPE-XA-08 treatment) showed lower GGT level when compared to the TAA control group (Group 3P) with value of 30.80 ± 3.980 IU/L mmol/L.

**Figure 1:**
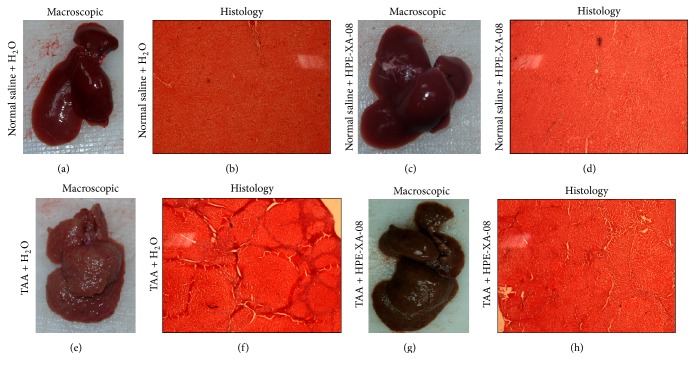
Macroscopic and histologic analysis of liver harvested from rats. Liver samples were harvested from rats inoculated with normal saline and treated with water (a), inoculated with normal saline and treated with HPE-XA-08 (c), inoculated with TAA (200 mg/kg) twice weekly and treated with water (e), and inoculated with TAA and treated with HPE-XA-08 (g). The sections from harvested liver were stained with hematoxylin-eosin, respectively (b, d, f, h).

Histological examination of liver section of rats from the control groups (Groups 1P and 2P) showed that the liver was dark red in color, with smooth homogenous surface texture (Figures 1(a) and 1(c)). The liver of rats from Group 3P was observed to be slightly brownish in color. The surface was rough, irregular with highly nodulated morphology (Figure 1(e)). In the rat group of TAA + HPE-XA-08 treatment (Group 4P), although the color of the liver was noted as dark brown, the surface of the liver was smoother and the nodules were not as distinct as the liver of rat group 3P (Figure 1(g)). The liver section of rats from control groups (Groups 1P and 2P) showed a homogenous distribution of hepatic cells devoid of extracellular matrix protein (ECM; Figures 1(b) and 1(d)). The liver section of TAA-treated control group (Group 3P) exhibited marked presence of ECM extending through the hepatic lobules and displayed as large fibrous septa (Figure 1(f)). However, no special histological staining was performed to determine the component of the septa. In the liver section of the TAA + HPE-XA-08 treatment group (Group 4P), the presence of ECM was almost not detected. The fibrous septa were not present as those observed in Figure 1(f), although trace of injury can still be observed (Figure 1(h)).

### 3.2. Treatment of TAA-Induced Liver Cirrhosis

**Figure 2:**
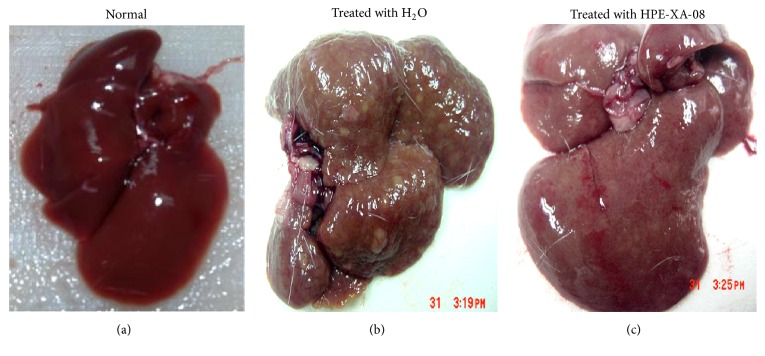
Macroscopic analysis of cirrhotic liver following treatment regime. Liver cirrhosis was induced by TAA for 12 weeks. After 12 weeks, the TAA was discontinued and rats were treated with either water (b) or HPE-XA-08 (c). Normal rat was included as negative control and treated with water (a).

The possibility for HPE-XA-08 to reverse liver cirrhosis in rats was also evaluated. All rats were inoculated with TAA to induce liver cirrhosis, except for rat group 1T which was inoculated with normal saline to serve as control group (normal rat). Following 12 weeks of induction of liver cirrhosis with TAA, the rats were either treated with distilled water (Group 2T) or with 600 mg/kg HPE-XA-08 for 30 days (Group 3T). The hepatic biochemical activities in normal and cirrhotic rats were evaluated before and after the treatment regime. The hepatic enzyme levels of ALT, AST, ALP, and GGT were measured (Table 2). It was noted that the AST, ALP, and GGT levels decreased after the 30-day treatment in all rat groups. The ALT level, however, was noted to increase in the cirrhotic rat groups (Groups 2T and 3T) following the 30-day treatment with increment from 62 ± 0.636 IU/L to 75 ± 0.577 IU/L in Group 2T and 63 ± 1.202 IU/L to 69 ± 0.441 IU/L in Group 3T. Withdrawal of TAA helped to significantly reduce the level of ALP and GGT in Group 2T although the rats were treated with just water. In rats treated with HPE-XA-08 (Group 3T), further decrease of ALP and GGT levels with reduction from 368 ± 0.882 IU/L to 102 ± 2.333 IU/L and 39 ± 0.296 IU/L to 15 ± 0.441 IU/L for ALP and GGT, respectively, was observed. The AST level was reduced significantly following treatment with HPE-XA-08 with reduction from 179 ± 0.882 IU/L to 158 ± 0.882 IU/L. Unlike those treated with HPE-XA-08, the AST level in the cirrhotic rat treated with water was 180 ± 1.155 IU/L and 175 ± 0.667 IU/L before and after treatment, respectively. Macroscopic observation of the liver from the normal control rat (Group 1T) showed the liver to be dark red in color with smooth homogenous surface (Figure 2(a)). In the cirrhotic control group treated with water (Group 2T), although the TAA has been discontinued, the liver was slightly brownish with macronodular structure and irregular nonhomogenous surface (Figure 2(b)). For the cirrhotic rats treated with HPE-XA-08, the color of the liver was more reddish compared to the group treated with only water (Figure 2(c)). The liver surface showed no obvious nodular structure and the surface of the liver was comparable to the normal liver.

## 4. Discussion

Hepatic cirrhosis, a consequence of hepatic fibrosis, is characterized by exaggerated production of extracellular matrix properties (ECM) [12]. Hepatic cirrhosis induced by thioacetamide (TAA) lead to steatosis, which is associated with the aggravation of lipid peroxidation and depletion of antioxidant status [13].

In the present study, liver damage is manifested by increases in serum ALP, GGT, and bilirubin levels. TAA-induced liver cirrhosis, however, was usually not characterized by high serum ALT and AST, unlike those intoxicated by CCl_4_ and paracetamol [14, 15]. Further, it has been reported that the severity of liver injury does not correlate with the level of liver enzyme elevation [16]. From our observation on the preventive effects of HPE-XA-08 on liver cirrhosis, although the differences between the level of liver enzymes in those untreated and those treated with HPE-XA-08 were not significant, except for level of GGT, the macroscopic and histologic examinations supported that treatment with HPE-XA-08 protected the liver against severe damage. Based on our findings, ALP and GGT could be employed as indicator in evaluating liver damage induced by TAA; however, the enzyme levels may not corroborate with the physical condition of the liver. The histological observation of the liver of rats given TAA was consistent with that reported by Bruck et al. [12], where increase in ECM was noted. In this study, however, we did not determine the mechanisms of action of the herbal concoction (HPE-XA-08) in providing the protective effects on the liver against induced damage. We postulated that the HPE-XA-08 decoction may affect the collagen content in the liver by either increasing the collagen synthesis, or reducing the collagen degradation, which was evidenced by the resolution of the fibrotic lesion and ECM in the liver of HPE-XA-08-treated rat observed macroscopically and histologically. The liver harvested from cirrhotic rat treated with HPE-XA-08 showed accelerated resolution of existing fibrosis. The morphology of the liver has reverted to almost comparable to the normal liver (Figure 2(c)). This finding was consistent with reports by Chen et al. [17] and Park et al. [18] that demonstrated that 2 out of 10 components within HPE-XA-08 and* Gardenia jasminoides *and* Scutellaria baicalensis* play a role in attenuation of collagen accumulation and apoptosis of hepatic stellate cells (HSCs), respectively, which help in resolution of liver fibrosis. Other than that, the resolution of liver damage may also be assisted by extract of* Ligustrum lucidum* which was reported to induce apoptosis and cell senescence in human hepatocellular carcinoma [19].

Liver fibrosis and cirrhosis were earlier thought of as irreversible processes. However, recent clinical and experimental evidences suggest that the process can be reversed [2, 20]. To assess the potential curative effects of HPE-XA-08 on liver cirrhosis, the hepatotoxic TAA was removed during the treatment process. We noted that although TAA has been withdrawn for 30 days, the liver nodulation could still be observed (Figure 2(b)); however, the liver condition was not as nodulated as observed in the liver with the presence of TAA (Figure 1(e)) suggesting possible arrest of progressive liver damage. Previous report showed that TAA-induced steatosis was due to accumulation of lipids within the hepatocytes [21]. Our observation in the rats' cirrhotic liver was consistent with this report (Figure 2(b)), where lipid nodules could be observed in the cirrhotic liver of rats treated with water (H_2_O). Amelioration of hyperlipidemic liver observed in our study could be contributed by constituents present in* Chrysanthemum morifolium* and* Artemisia scoparia* (within HPE-XA-08 decoction), which has been described to attenuate high-fat milk-induced fatty liver and reduce the liver lipid accumulation, respectively [22, 23]. In the assessment of liver enzyme, the level obtained before the treatment was used as a baseline to be compared to the enzyme level after the treatment. It was noted that the levels of liver enzyme in normal rat decreased after 30 days of treatment with water. This could be related to the age of the rat, since age does affect the liver enzymes [24], which indicated that observation of liver enzyme level alone was insufficient in evaluating hepatoprotective effects conferred by any herbal supplement and needed to be complemented with biopsy or histology.

Most of the previous studies on the antihepatofibrotic were performed on single plant/herb extract. In this study, the hepatocellular injury and fibrosis in rats were treated orally with decoction consisting of 10 medicinal Chinese herbs, named HPE-XA-08. Other than the 5 plants/herbs within HPE-XA-08 that have been mentioned above, the remaining 4 plants/herbs in the decoction,* Phellodendron amurense*,* Poria cocoa*,* Bupleurum B. scorzoneraefolium,* and* Tarxacum mongolicum,* scientifically were reported to exert anticarcinogenic and antitumor properties [25–28]. Based on our findings, we conclude that the decoction consisting of the 10 medicinal Chinese herbs, named HPE-XA-08, possesses potential hepatoprotective and curative effects against damaged liver. Further investigations, however, are needed to identify the hepatoprotective mechanisms conferred by this aqueous herbal preparation. The present finding provides scientific evidence that the mixture of these 10 Chinese traditional herbs preparation possessed ethnomedicinal properties in maintaining liver function and could be developed further as ethnomedicine for liver injuries.

## Figures and Tables

**Table 1:** Effect of HPE-XA-08 on blood biochemicals related to liver damage (ALT, AST, ALP, and GGT) for observation of protective activity.

Group	ALT (IU/L)	AST (IU/L)	ALP (IU/L)	GGT (IU/L)
Normal saline + H_2_O (1P)	80.00 ± 4.175	237.3 ± 12.84	173.6 ± 7.807	2.286 ± 0.4206
Normal saline + HPE-XA-08 (2P)^#^	76.00 ± 4.416	223.3 ± 11.16	170.3 ± 23.68	3.750 ± 0.4787
TAA + H_2_O (3P)	88.50 ± 12.75	215.0 ± 13.64	375.0 ± 10.41^*∗*^	37.50 ± 1.857^*∗*^
TAA + HPE-XA-08 (4P)^+^	69.40 ± 7.756	222.4 ± 5.715	277.0 ± 4.722	30.80 ± 3.980

^*∗*^Levels of enzymes between 1P and 3P were compared to show the effects of TAA on the level of liver enzymes; *∗* indicates *p* < 0.05.

^#^Levels of enzymes between 1P and 2P were compared to show the effects of HPE-XA-08 on the level of liver enzymes but did not show statistically significant value (*p* > 0.05).

^+^Levels of enzymes between 3P and 4P were compared to show the effects of HPE-XA-08 on preventing TAA-induced liver cirrhosis but did not show statistically significant value (*p* > 0.05).

**Table 2:** Blood enzymes related to liver damage (ALT, AST, ALP, and GGT) for observation of curative activity conferred by HPE-XA-08.

Liver enzyme	Normal rat (Group 1T)		Cirrhotic rat			
			Treated with distilled water (Group 2T)		Treated with 600 mg/kg HPE-XA-08 (Group 3T)	
	Concentration of liver enzyme (before treatment; IU/L)	Concentration of liver enzyme (after treatment; IU/L)	Concentration of liver enzyme (before treatment; IU/L)	Concentration of liver enzyme (after treatment; IU/L)	Concentration of liver enzyme (before treatment; IU/L)	Concentration of liver enzyme (after treatment; IU/L)
Alanine aminotransferase (ALT)	77 ± 1.528	64 ± 0.318^a^	62 ± 0.636^b^	75 ± 0.577^a,b^	63 ± 1.202^b^	69 ± 0.441^a,b,c^
Aspartate aminotransferase (AST)	203 ± 3.512	184 ± 1.856^a^	180 ± 1.155^b^	175 ± 0.667^b^	179 ± 0.882^b^	158 ± 0.882^a,b,c^
Alkaline phosphatase (ALP)	198 ± 1.856	96 ± 1.155^a^	368 ± 1.155^b^	126 ± 0.667^a,b^	368 ± 0.882^b^	102 ± 2.333^a,b,c^
G-Glutamyl transferase (GGT)	2 ± 0.333	1 ± 0.067	38 ± 0.333^b^	25 ± 0.577^a,b^	39 ± 0.296^b^	15 ± 0.441^a,b,c^

^a^*p* < 0.05 when comparing level before and after treatment.

^b^*p* < 0.05 when compared with normal control (Group 1T).

^c^*p* < 0.05 when compared with untreated cirrhotic group (Group 2T).

## List of Abbreviations

ALT: Alanine aminotransferase
ALP: Alkaline phosphatase
AST: Aspartate aminotransferase
ECM: Extracellular matrix
GGT: Gamma-glutamyl transferase
H_2_O: Water
IP: Intraperitoneal
SEM: Standard error of mean
TAA: Thioacetamide.
